# Independent Risk Factors and Mortality Implications of De Novo Central Nervous System Involvement in Patients Hospitalized with Severe COVID-19: A Retrospective Cohort Study

**DOI:** 10.3390/jcm13133948

**Published:** 2024-07-05

**Authors:** Andreea Raluca Hanganu, Adriana Octaviana Dulămea, Cristian-Mihail Niculae, Emanuel Moisă, Adriana Hristea

**Affiliations:** 1 Faculty of Medicine, University of Medicine and Pharmacy “Carol Davila”, 050474 Bucharest, Romania; andreea.florea@drd.umfcd.ro (A.R.H.); cristian.niculae@drd.umfcd.ro (C.-M.N.); emanuelmoisa@gmail.com (E.M.); adriana.hristea@umfcd.ro (A.H.); 2 National Institute for Infectious Diseases “Prof. Dr. Matei Bals”, 021105 Bucharest, Romania; 3 Fundeni Clinical Institute, 022328 Bucharest, Romania; 4 Elias University Emergency Hospital, 011461 Bucharest, Romania

**Keywords:** severe COVID-19, SARS-CoV-2, central nervous system, risk factors, outcome, mortality

## Abstract

**Background/Objectives**: Central nervous system (CNS) involvement is a complication of COVID-19, adding to disease burden. The aim of this study is to identify the risk factors independently associated with CNS involvement in a cohort of patients hospitalized with severe forms of COVID-19 and the risk factors associated with all causes of in-hospital mortality and assess the impact of CNS involvement on in-hospital mortality of the severe COVID-19 patients. **Methods**: We performed a retrospective observational cohort study including adult patients with severe or critical forms of COVID-19 with and without new-onset CNS manifestations between March 2020 and December 2022. **Results**: We included 162 patients, 50 of which presented with CNS involvement. Independent risk factors for CNS involvement were female sex (*p* = 0.04, OR 3.67, 95%CI 1.05–12.85), diabetes mellitus (*p* = 0.008, OR 5.08, 95%CI 1.519–17.04)), lymphocyte count (0.04, OR 0.23, 95%CI 0.05–0.97), platelets count (*p* = 0.001, OR 0.98, 95%CI 0.98–0.99) CRP value (*p* = 0.04, OR 1.007, 95%CI 1.000–1.015), and CK value (*p* = 0.004, OR 1.003, 95%CI 1.001–1.005). Obesity was a protective factor (*p* < 0.001, OR 0.57, 95%CI 0.016–0.20). New-onset CNS manifestations (*p* = 0.002, OR 14.48, 95%CI 2.58–81.23) were independent risk factors for in-hospital mortality. In-hospital mortality was higher in the new-onset CNS involvement group compared to patients without neurological involvement, 44% versus 7.1% (*p* < 0.001). **Conclusions**: CNS involvement in severe COVID-19 patients contributes to all causes of in-hospital mortality. There are several risk factors associated with new-onset CNS manifestations and preventing and controlling them could have an important impact on patients’ outcome.

## 1. Introduction

Central nervous system (CNS) involvement is a well-known complication of COVID-19, adding to the overall disease burden [1,2,3]. Since the beginning of the pandemics, an array of neurological manifestations has been reported, ranging from mild symptoms such as anosmia/ageusia, headache, and myalgias, to more severe ones like encephalopathy, neurovascular events, epilepsy, and encephalitis [4]. The prevalence of neurological manifestations among hospitalized patients with COVID-19 varies up to 80% with clinically patent syndromes being associated with a higher risk of in-hospital death [5].

Although neurological involvement in SARS-CoV-2 infection has been largely described, there are limited data regarding independent risk factors for CNS involvement in severe COVID-19 patients, as the majority of research focuses on COVID-19-related stroke [6,7,8] or does not stratify for COVID-19 severity [1].

In this study, we aim to identify the risk factors associated with CNS involvement in a cohort of patients hospitalized with severe forms of COVID-19. Second, we aim to identify the risk factors associated with all causes of in-hospital mortality and describe the impact of CNS involvement on in-hospital mortality of severe COVID-19 patients.

## 2. Materials and Methods

### 2.1. Study Design and Participants

**Figure 1:**
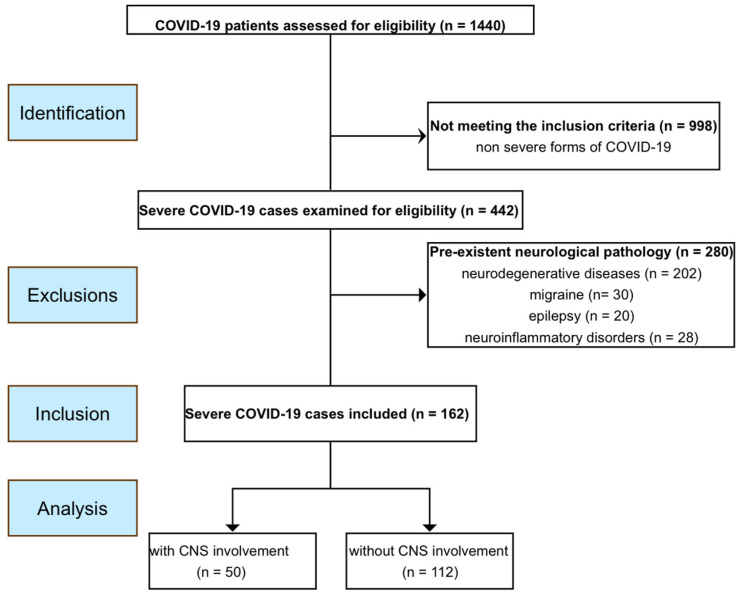
Flowchart of the study: recruitments, inclusions, and exclusions.

We performed a retrospective observational cohort study including adult patients (>18 years old) with severe or critical forms of COVID-19 hospitalized in a tertiary infectious diseases hospital between March 2020 and December 2022. Patients were divided into two study groups: (i) patients with CNS involvement and (ii) patients without neurological involvement. The patients with neurological involvement have all been examined by the same neurologist established to tend to patients hospitalized with COVID-19 infection in the hospital unit. We excluded patients with preexistent neurological chronic diseases such as chronic migraine, epilepsy, neuroinflammatory disorders, and neurocognitive disorders. The flowchart of the study is presented in Figure 1. This study was approved by the local Bioethics committee (C13303/07.11.2023).

### 2.2. Data Collection

We reviewed the physical and electronic medical records of the patients and collected data regarding demographics (gender, age), medical history and comorbidities (arterial hypertension, ischemic heart disease, stroke history, diabetes mellitus, chronic kidney disease), laboratory workup at admission (complete blood count, C-reactive protein, creatinine, lactate dehydrogenase, creatine kinase, liver enzymes, INR, D-dimers), length of stay, and outcome. No follow-up data post-discharge were available.

### 2.3. Definitions

Neurological data were extracted from clinical exams and diagnoses established by the neurologist. The following diagnoses were considered as new-onset CNS involvement: COVID-19-associated encephalopathy, neurovascular events, seizures, and COVID-19-associated headache. COVID-19 encephalopathy was defined as new-onset cognitive decline or alteration of the consciousness without other identified causes. COVID-19 headache was defined as a headache with unusual characteristics, unrelated to fever, hypoxia, or other causes. SARS-CoV-2 infection was considered severe based on at least one of the following criteria: peripheral oxygen saturation ≤93% in ambient air, respiratory rate >30/minute, arterial oxygenation partial pressure to fractional inspired oxygen ratio <300, or lung infiltrates >50% of lung parenchyma, using the World Health Organization COVID-19 severity criteria. Obesity was defined as a general term comprised of all types of obesity, including overweight patients.

### 2.4. Statistical Analysis

Categorical data were presented as absolute (number) and relative frequencies (%). Continuous variables following a normal distribution were expressed as mean ± Standard Deviation (SD), while continuous variables with non-normal distribution were expressed as median (range). The normal distribution of variables was assessed by using the Shapiro–Wilk test. Normally distributed continuous variables were compared using an independent *t*-test, while continuous variables that deviated from normal distribution were compared using the Mann–Whitney U test. The chi-square test (or Fisher’s exact test) was used to compare categorical variables between groups.

Binary logistic regression was used to assess the predictive value of different variables regarding factors associated with CNS manifestations development in patients with severe COVID-19. A stepwise (backward likelihood ratio) method was used for variable selection in the final model. The rationale for introducing the variables in the model was based both on basic inference analysis and clinical judgment taking into consideration data reported in the medical literature. The presence of CNS involvement was considered the dependent variable, while the following were introduced as independent variables: age, gender, obesity, arterial hypertension, ischemic heart disease, history of stroke, chronic kidney disease, absolute number of leukocytes, lymphocytes and platelets, hemoglobin value, C-reactive protein, LDH, creatinine, AST, D-dimers, and INR value. Furthermore, collinearity diagnostics were performed and none of the variables had a variance inflation factor >1.5. The model’s goodness-of-fit was evaluated through the Hosmer–Lemeshow test. Variables were kept in the final model if *p* < 0.05 and removed if *p* > 0.1. Nagelkerke’s R square value and the percentage of cases correctly predicted were also reported. Lastly, a similar predictive model was performed for all-cause hospital mortality.

All tests were two-tailed and were considered significant if *p* < 0.05. Statistical analysis was performed using Statistical Package for Social Sciences (SPSS version 28, IBM Corp., Armonk, NY, USA).

## 3. Results

### 3.1. Baseline Characteristics of Patients with Severe COVID-19

During the study period, we revised 1440 files from hospitalized patients based on the severe COVID-19 diagnostic. Of these, we included 162 patients, 50 of which presented with CNS involvement. The cohort was composed of 112 (69.1%) males. The mean age for the whole group was 60.75 ± 14.35 years. In terms of medical history, 87 (53.7%) patients had high blood pressure, 39 (24.1%) had diabetes mellitus, 9 (5.6%) had ischemic heart disease, 108 (66.7%) were overweight or obese, 11 (6.8%) had a history of stroke, and 8 (4.9%) had chronic kidney disease. Median (range) CRP was 39.9 (0.16–345) mg/L. WBC, lymphocytes, platelets, and hemoglobin had medians (range) of 8.35 (1.7–40.5) N × 10^3^/μL, 0.9 (0.1–3.3) N × 10^3^/μL, 256.75 (13.0–650.0) N × 10^3^/μL, and 13.5 (1.6–17.5) mg/dL, respectively. Median (range) D-dimers were 278.5 (5.4–36937.0) pg/mL and median (range) LDH was 380.0 (149.0–5399.0) U/L. Median (range) values for creatinine, creatinine kinase, alanin aminotransferase, and aspartate aminotransferase were 0.9 (0.3–5.2) mg/dL, 69 (20.0–2567.0) U/L, 47.0 (14.0–4990.0) U/L, and 49.0 (14.0–7102.0) U/L, respectively.

### 3.2. Central Nervous System Manifestations

The most common CNS manifestation was COVID-19 encephalopathy in more than half of patients, followed by neurovascular events. Of the 15 patients who presented neurovascular events, 13 had an ischemic stroke, 1 had a concomitant ischemic stroke and venous thrombosis, and 1 had an ischemic stroke that transformed hemorrhagically. Other neurovascular events were hemorrhagic stroke in 5 patients, subarachnoid hemorrhage in 1 patient, and transient ischemic attack in 1 patient. CNS manifestations are presented in Table 1.

### 3.3. Factors Associated with CNS Involvement in Patients with Severe COVID-19

Compared to patients without neurological involvement, patients with neurological manifestations were predominantly females, older, and had cardiovascular risk factors (history of ischemic heart disease, diabetes mellitus, high blood pressure). The median duration of hospitalization was 25 (6–65) days for patients with CNS involvement and 14 (6–44) days in patients without neurological involvement. CRP and D-dimer values were higher in the neurological involvement group, and lymphocytes, platelets, and hemoglobin were lower. The obesity rate was higher in the group without neurological involvement. Cohort characteristics are summarized in Table 2.

After performing binary logistic regression, the following variables were identified to be independently associated with CNS involvement in severe COVID-19 patients: female sex (*p* = 0.04, OR 3.67, 95%CI 1.05–12.85), diabetes mellitus (*p* = 0.008, OR 5.088, 95%CI 1.519–17.04), lymphocyte count (0.04, OR 0.23, 95%CI 0.05–0.97), platelets count (*p* = 0.001, OR 0.98, 95%CI 0.98–0.99), CRP value (*p* = 0.04, OR 1.007, 95%CI 1.000–1.015), and CK value (*p* = 0.004, OR 1.003, 95%CI 1.001–1.005). Obesity was a protective factor for CNS involvement (*p* < 0.001, OR 0.057, 95%CI 0.016–0.200). The results are reported in Table 3.

### 3.4. CNS Involvement as an Independent Risk Factor for In-Hospital Mortality in Severe COVID-19 Patients

Age was an independent risk factor for in-hospital mortality (*p* = 0.03, OR 1.06, 95%CI 1.006–1.120). New-onset CNS manifestations (*p* = 0.002, OR 14.48, 95%CI 2.58–81.23) and personal history of neurovascular events (*p* = 0.007, OR 12.74, 95%CI 1.99–81.31) were also independently associated with in-hospital mortality risk factors. Laboratory variables associated with in-hospital mortality were WBC count (*p* = 0.009, OR 1.12, 95%CI 1.03–1.24), platelets count (*p* = 0.002, OR 0.98, 95%CI 0.98–0.99), and LDH (*p* = 0.011, OR 1.003, 95%CI 1.001–1.005). The results are reported in Table 4.

## 4. Discussion

In this study, we identified the independent risk factors for new-onset CNS manifestations in patients with COVID-19 as being female sex, diabetes mellitus, lymphopenia, thrombocytopenia, and high titers of CRP and CK.

Female sex has previously been identified as an independent risk factor for the development of long COVID-associated neuropsychiatric disorders, such as anxiety and depression, but until now there is no evidence that women infected with SARS-CoV-2 have higher rates of acute neurological complications [9,10]. In a one-year prospective study following patients who suffered from mild COVID-19, female patients reported more frequent headaches, hyposmia, and cognitive deficits [11]. The reason for these findings is still debatable, but the immunological response might play an important role in these sex-related differences [10]. Females are four times more predisposed to autoimmune diseases than men [12], and while the circumstances of this predisposition remain unclear, several theories emerged: B cell activation was more enhanced in women, producing more antibodies, thus increasing the risk for autoimmune diseases [12]; the influence of the sex hormones (estrogens, progesterone, and testosterone) over the immune system [13]; and the differences in gut microbiota composition between males and females [14]. Further research is needed to confirm the female predisposition to acute CNS complications in severe COVID-19 and to better understand the possible mechanism.

Diabetes mellitus is a known factor for an array of neurological complications even outside of COVID-19. Several authors reported that diabetes mellitus is an independent risk factor for severe COVID-19 and mortality in COVID-19 patients [15,16]. In a meta-analysis by Radwan et.al., diabetes together with dyslipidemia and heart disease doubled the risk of neurological complications in patients with COVID-19, without mentioning the disease severity. In our cohort, almost 60% of patients with diabetes and severe COVID-19 had CNS involvement, confirming diabetes mellitus as an independent risk factor for the development of new CNS manifestations in patients with severe COVID-19.

Lymphopenia is an independent risk factor for severe forms of COVID-19, and the decreasing trend of lymphocytes following hospitalization increases the risk of death [17]. Furthermore, immunodeficiencies can be associated with a variety of neurological manifestations [18]. However, data regarding the impact of lymphopenia on developing neurological manifestations in SARS-CoV-2 infected patients are scarce. In a study performed by Li et.al., lower lymphocyte count, indicating immunosuppression, together with higher CRP titer were associated with a higher risk of cerebrovascular diseases [3]. In a study reporting neurological manifestations in COVID-19 taking place in Iran, the median lymphocyte count was also significantly lower in patients with neurological manifestations [19].

Another biomarker that proved to be an independent risk factor for new-onset neurological manifestations in severe COVID-19 is thrombocytopenia. Although medians of the thrombocytes in both groups were in the normal range, lower counts of platelets are an independent risk factor for CNS involvement. In another study, there was no difference between patients with and without neurological manifestations regarding platelet count [19]. The association between COVID-19 cerebrovascular events and thrombocytopenia could be a result of both autoimmune and vascular mechanisms. There are numerous reports of induced thrombocytopenia due to anti-SARS-CoV-2 vaccine and concomitant stroke [20,21,22]. In a meta-analysis, thrombocytopenia was significantly associated with severe COVID-19 [23]. In our study, platelets were independent risk factors for mortality as well. Additional data are needed to assess if the link between low platelet count and CNS manifestations is direct, or due to the immunological mechanisms of the cytokine storm in severe forms of COVID-19, especially considering they are also a marker for poor outcome.

High values for CRP and CK were also identified as independent risk factors for new CNS manifestations. CRP is a direct marker of inflammation and of the cytokine storm in SARS-CoV-2 infection [24]. Our results emphasize once more the impact inflammation has on the nervous system. Elevated levels of CRP in patients with neurological manifestations were reported also by Ashrafi et al. [19]. The various CNS manifestations, from headache to stroke, seizures, and encephalopathy, are the result of enhanced permeability of the blood–brain barrier together with the formation of microthrombi [25,26].

CK values usually rise in stroke, with high titer being a marker of recurrence, death, and disability, although the source tissue remains controversial [27]. In a previous study regarding CK in COVID-19, high CK was associated with disease severity, even when adjusted for CRP values, without association with skeletal muscle symptoms [28]. However, in a meta-analysis, a link between SARS-CoV-2 myopathy and CK levels was observed [23]. Nevertheless, what both authors agree on is the association between CK levels and disease severity [23,28].

As mentioned, obesity was surprisingly identified as a protective factor for new CNS manifestations in severe COVID-19 patients. Several studies reported a link between obesity and severe forms of COVID-19 [29,30,31]. However, although the risk of admission to the ICU is higher in obese patients, some authors reported no difference in mortality rate between obese and non-obese patients [32,33]. Furthermore, in a cohort of patients with type 2 diabetes mellitus and COVID-19, obesity was associated with 7-day mortality only in patients under 75 years old, while in patients older than 75 years no such association was observed [34]. These data are in agreement with Tartof et al. who reported obesity as an independent risk factor for COVID-19 mortality especially in young male patients, even after adjusting for obesity-associated comorbidities [35]. Lighter et al. lowered the threshold for obesity being an independent risk factor for ICU admission to 60 years old [36]. In our study, the mean age for the full cohort was 60.75 ± 14.35, and 68.3 ± 13.49 for patients with neurological involvement; thus, the patients with neurological involvement were older than the cohort mean. Advanced age might be an explanation for the protective role of obesity over neurological events, but more detailed research, such as case-control studies, is needed to define the causality of this relationship.

In another study on ischemic stroke risk factors in COVID-19 patients, higher BMI and obesity proved to be protective factors [7]. The obesity paradox in stroke is also a studied phenomenon outside of COVID-19, but it still remains a controversial entity as most data are observational. We want to emphasize the fact that although obesity proved to be a protective factor for CNS manifestation, it has no influence over in-hospital mortality. At this point, one can only speculate the causes of this paradox: perhaps obese patients with severe COVID-19 have more resources to tolerate prolonged ICU stays or critical health states, or they might also follow treatment for various comorbidities associated with obesity, such as antiplatelets, anticoagulants, statins, etc. Further, more detailed studies, on larger cohorts, are needed in order to assess the existence of a true obesity paradox in CNS-related COVID-19 manifestations.

New-onset CNS manifestations and personal history of neurovascular events were identified as independent risk factors for in-hospital mortality in COVID-19 patients. Similar findings were described by various authors [1,2,37,38]. Moreover, in an article by Eskandar et al., CNS manifestations such as altered mental state or stroke predicted mortality independent of COVID-19 severity [2]. Ischemic stroke was identified as being the most frequent neurological complication linked to mortality [38]. In another study, all neurological manifestations were associated with higher odds of death [39]. Other independent risk factors for in-hospital mortality were high WBC, low platelets count, and high LDH values.

This study has a few limitations. First of all, its retrospective nature restricted the collection of enough data regarding oxygen therapy, BMI, other inflammation markers, and the dynamics of biological markers corroborated with the clinical picture and onset of neurological manifestations. Furthermore, there were logistic limitations regarding imagistic investigations and CSF analysis data during pandemics. The lack of follow-up data is another limitation as the long-term impact of CNS involvement in severe COVID-19 patients could be valuable. Moreover, another limitation is the fact that patients come from the same geographic area and the lack of racial diversity, thus assumptions for different populations cannot be made.

In conclusion, CNS involvement in COVID-19 patients is an important complication, contributing to in-hospital mortality and overall disease burden.

## Figures and Tables

**Table 1:** Spectrum of CNS manifestations in severe COVID-19 patients.

Type of CNS Involvement	Number of Patients N = 50
COVID-19-associated encephalopathy, N (%)	38 (76)
Neurovascular events, N (%)	22 (44)
New-onset epileptic seizures, N (%)	7 (14)
COVID-19-associated headache, N (%)	7 (14)

**Table 2:** Cohort demographics and laboratory characteristics.

		With Neurological Involvement N = 50	Without Neurological Involvement N = 112	*p*
Female, N (%)		22 (44)	28 (25)	0.018
Age (mean ± STD)		68.3 (±13.49)	57.38 (±13.46)	<0.001
CVD risk factors	HBP N (%)	37 (74)	50 (44.6)	0.001
	Diabetus mellitus N (%)	23 (46)	16 (14.3)	0.000
	Ischemic heart disease, N (%)	6 (12)	3 (2.7)	0.025
	Obesity/overweight N (%)	13 (26)	95 (84.8)	<0.001
Lab (Median, range)	WBC (N × 10^3^/μL)	6.6 (2.2–20.44)	8.35 (1.7–40.5)	0.24
	Lymphocytes (N × 10^3^/μL)	0.7 (0.3–1.49)	1 (0.1–3.3)	0.000
	Platelets (N × 10^3^/μL)	172 (83–314)	290.5 (13–650)	0.000
	Hemoglobin (mg/dL)	13.1 (6.92–15.58)	13.75 (1.6–17.5)	0.001
	CRP (mg/L)	76.7 (1.5–302)	35.3 (0.16–345)	0.000
	D-Dimers (pg/mL)	389 (5.4–5471)	242 (36–6937)	0.000
	CK (U/L)	138 (20–1167)	53 (20–2567)	0.005
	LDH (U/L)	397.34 (222–1872)	368.5 (149–5399)	0.137
	Creatinin (mg/dL)	1 (0.3–3.2)	0.8 (0.4–4.4)	0.183
	ALT (U/L)	39 (17–194)	54.5 (16–4990)	0.002
	AST (U/L)	51 (29–151)	48.5 (20–7102)	0.733
Hospitalization duration (days), median (range)		25 (6–65)	14 (6–44)	0.000
In-hospital all-cause mortality N (%)		22 (44)	8 (7.1)	<0.001

**Table 3:** Independent risk factors for CNS involvement (method: stepwise backward likelihood ratio, *p* for model <0.01, Nagelkerke R square = 0.69, Hosmer–Lemeshow test, chi-square = 4.29, *p* = 0.89, % cases correctly predicted = 88.6%).

	*p*	OR	95%CI	
			Lower	Upper
CKD	0.063	6.513	0.900	47.107
Diabetes mellitus	0.008	5.088	1.519	17.040
Obesity	<0.001	0.057	0.016	0.200
Female sex	0.042	3.672	1.049	12.847
Lymphocytes	0.046	0.227	0.053	0.972
Platelets	<0.001	0.989	0.982	0.995
CRP	0.046	1.007	1.000	1.015
Constant	0.006	77.945		

**Table 4:** Independent risk factors for in-hospital mortality (method: stepwise backward likelihood ratio, *p* for model <0.01, Nagelkerke R square = 0.671, Hosmer–Lemeshow test, chi-square = 1.61, *p* = 0.99, % cases correctly predicted = 88.9%).

	*p*	OR	95% CI	
			Lower	Upper
Age	0.030	1.061	1.006	1.120
Stroke history	0.007	12.739	1.996	81.317
WBC	0.009	1.132	1.032	1.242
Platelets	0.002	0.988	0.981	0.996
LDH	0.011	1.003	1.001	1.005
CNS involvement	0.002	14.482	2.579	81.323
Constant	0.001	0.001		

## Data Availability

All data are available upon request addressed to the corresponding author.
